# Childhood Hodgkin Lymphoma in Sub-Saharan Africa: A Systematic Review on the Effectiveness of the Use of Chemotherapy Alone

**DOI:** 10.1177/2333794X231223266

**Published:** 2024-01-05

**Authors:** Hellen Mugarra Kabahweza, Angela Spencer

**Affiliations:** 1 University of Manchester, Manchester, UK; 2 Joint Clinical Research Center, Kampala, Uganda

**Keywords:** hematology/oncology, Hodgkin lymphoma, chemotherapy, SubSaharan Africa

## Abstract

*Introduction.* Childhood Hodgkin lymphoma (HL) is often curable, but in Sub-Saharan Africa (SSA), access to standard treatments like combined chemotherapy and radiotherapy is limited. This study aimed at evaluating the effectiveness of using chemotherapy alone for children with HL in SSA. *Methods.* We searched Medline, Embase, Cinahl Plus and Cochrane Central databases for records of studies that evaluated childhood HL survival outcomes from January 2000 to December 2022. *Results.* Six observational studies were identified for inclusion, and 3 were included in the meta-analysis. Most HL cases included in the analysis presented with lymphadenopathy and the nodular sclerosing subtype, with a high percentage (80%) in advanced stages. The GRADE certainty of the evidence for the assessed outcomes was rated as very low. Overall survival with chemotherapy was 67.8% (95% CI: 42.1%-88.8%). *Conclusion.* Chemotherapy is a potential treatment choice for childhood HL in SSA. However, it is crucial to approach this option cautiously due to the limited certainty of the supporting evidence. To improve outcomes for affected children in SSA, more robust studies are needed, along with a focus on early detection and supportive care.

## Introduction

Hodgkin lymphoma (HL) is one of the main types of lymphoma.^1,2^ HL is a B-cell lymphoma characterized by the presence of atypical, large cells called Reed-Sternberg cells.^3^ Worldwide, HL is an uncommon form of lymphoma that contributes to 6% of childhood cancers,^2,4^ 0.4% of all new tumors, and 0.3% of all cancer deaths,^5^ with an incidence of 1.2 per 100,000 children in high-income countries.^3^ HL represents the most prevalent cancer in children,^3^ and incidence varies with age, gender, and setting. Globally, HL is the most common type of cancer among patients aged 15 to 19 years yet commonest in children under 10 years old in the developing countries.^2,5^ In Africa, the incidence rate for HL has been challenging to estimate due to the unreliable and limited cancer registries in the region.^6^

The epidemiology of HL in Africa is greatly influenced by the high rate of infectious diseases like HIV, endemic malaria, Epstein-Barr virus (EBV), and herpes virus, which contribute to increased morbidity and mortality (a reduced survival rate) in the region compared to resource-rich countries.^7^ With chemotherapy-only treatment, over 90% of children with HL in stages I and II, and 70% of those in stages III and IV, survive in developed countries,^8,9^ compared to 64% to 71% survival with chemotherapy-alone treatment in Africa.^10,11^

The recommended treatment for HL in children is chemotherapy and radiotherapy.^12,13^ However, many countries in SSA use chemotherapy alone (CT) to manage childhood HL due to a lack of or inadequate radiation therapy services.^10^ In SSA, 25 countries have no access to radiotherapy treatment, including countries such as Burundi, Malawi, Lesotho, and Chad. In some countries with large populations, like Nigeria and Ethiopia, approximately 5 million people rely on one radiotherapy machine. Whereas in other countries like Madagascar and Cameroon, radiotherapy use is declining.^14^

In SSA, a study using Adriamycin, bleomycin, vincristine and dacarbazine (ABVD) chemotherapy alone showed a 1-year survival rate of 67.5% and a 30% mortality rate.^11^ In developed countries, the use of mechlorethamine, vincristine, procarbazine, and prednisolone (MOPP) chemotherapy alone achieved a much higher event-free survival rate of 90%.^8^

While studies in developed countries have shown improved overall survival with chemotherapy plus radiotherapy for early-stage HL, the use of combined therapy is decreasing even in the United States.^15^ Developed countries use various radiotherapy techniques to minimize late effects,^15,16^ but the utilization of radiotherapy remains low in SSA.^14^ Common chemotherapy-only regimens in SSA include ABVD^11^ and Cyclophosphamide, Oncovin, Procarbazine, Prednisone, Adriamycin, Bleomycin, and Vinblastine (COPP-ABV).^3,17^

Despite the prevalence of childhood HL in SSA,^18^ there exists a notable gap in the literature regarding comprehensive studies that specifically investigate the outcomes of chemotherapy and radiotherapy in children. The majority of existing research predominantly focuses on the adult population and developed countries, leaving a knowledge gap in understanding the disease’s behavior and treatment responses in the context of SSA. This paper assesses the effectiveness of treating childhood HL with chemotherapy alone on survival in SSA.

## Materials and Methods

This systematic review was conducted in line with the preferred reporting items for systematic reviews and meta-analyses (PRISMA) guidelines.^19^ The protocol was registered in PROSPERO with registration ID CRD42023394080.

### Ethical Approval and Informed Consent

This was not needed since it was a review of published studies.

### Search Strategy

A systematic search of databases was carried out using medical subject headings (MeSH) in MEDLINE and the Cochrane central register of controlled trials (CENTRAL), free text words in Cinahl, and Emtree terms in Embase. For example, Medline was searched using the exploded MeSH terms “Hodgkin Disease” OR “Hodgkin Lymphoma” OR “Hodgkin’s Disease” OR “Hodgkin’s Lymphoma” OR “Hodgkins Lymphoma” OR “Hodgkins Disease” AND “Drug Therapy” OR “Drug Therap*” OR Chemotherap* AND “Africa South of the Sahara” and a search block for children (Supplemental Table S1). The same strategy was used in the other databases for all articles published from January 2000 to December 2022. Medline was searched via PubMed, Embase via Ovid, Cinahl Plus via EBSCO, and Cochrane Central via the Wiley Interscience interface. Boolean operators “AND” between concepts and “OR” within the concepts ensured the search was as comprehensive as possible.

### Eligibility Criteria

A PICOS (population, intervention, comparator, outcome, and study design) approach was developed as the eligibility criteria. **Population**: studies were eligible if they reported on children with Hodgkin lymphoma between 0 and 18 years in SSA. **Intervention**: treatment with chemotherapy only regimens. **Outcome**: these included overall survival, progression-free survival, event-free survival, relapse, and mortality. **Study design**: observational and intervention studies. We included Studies that published in English between January 2000 and December 2022. **Exclusion**: This included animal studies, conference abstracts, reviews, studies that used interventions other than chemotherapy alone, studies below case-control in the hierarchy of evidence, and studies that had an adult population. Preprints related to the review topic and references within the identified studies were reviewed to identify additional relevant studies. Reports were exported to the Covidence software^20^ which automatically removed duplicate and irrelevant articles. This was followed by tittle and abstract, then full text screening. Study selection was done by 2 independent reviewers (HMK and AS), and disagreements were resolved by consensus. Reviewers extracted data and completed the Microsoft excel sheet for all the studies that met the eligibility criteria.

### Survival Measures

Measures of survival considered in this review were: overall survival (the duration from the date of diagnosis to death or last follow-up,^21^ with no restriction on the cause of death); progression-free survival (when the patient’s signs and symptoms have resolved, as evidenced by tests done at the end of treatment)^22^; event-free survival (period after the first cancer treatment that the patient has not had the disease come back or worsen)^23^; complete remission (when the patient’s signs and symptoms have resolved, as evidenced by tests done at the end of treatment)^24^; mortality (the number of people who die in a given period having had cancer)^23^; and treatment abandonment (the failure to start/complete cancer therapy).^25^ These were expressed as proportions. For studies that had insufficient details available in their texts, the corresponding authors were contacted to obtain the required information.

### Quality Assessment

The risk of bias was assessed using the National Institute of Health (NIH) study quality assessment tool for observational cohort and cross-sectional studies.^26^ The GRADE tool^27,28^ was used to grade the quality of the evidence generated in this review across all outcomes and provided summaries of evidence that informed recommendations for clinical practice.

### Data Synthesis

The data in this review was presented according to study characteristics, participant characteristics, interventions used in the studies, and outcomes measured. Figures, tables, and free-text words were used to summarize and collate the data and evidence generated from the individual studies. Meta-analysis using version MedCalc statistical software version 20.110 was used to synthesize the findings from the included studies. The pooled proportion of survival outcomes was estimated across the included studies to obtain the overall effect size of the effectiveness of chemotherapy using the random-effect model. The Higgin’s *I*^2,29^and Egger’s^30^ tests were used to detect heterogeneity and publication bias respectively. Any study that was sought to have a high Risk of bias was excluded from the meta-analysis to limit invalid and unreliable results.

## Results

### Study Selection

In our analysis, we initially identified 381 records through a comprehensive search of the literature. Two hundred twenty-seven duplicate records were removed to avoid double-counting data from the same study, and then strict inclusion and exclusion criteria were applied to screen the 154 records.

**Figure 1.**
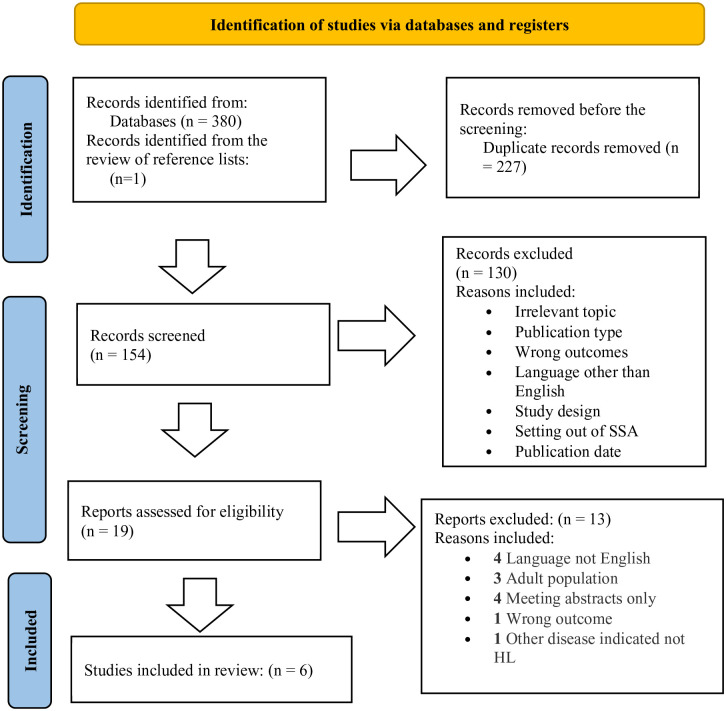
PRISMA flow diagram for study eligibility in this review.

A total of 6 full-text papers^3,17,31-33^ were included in the review. All studies included used chemotherapy-alone treatment regimens to manage childhood HL. The search process using the Preferred Reporting Items for Systematic Reviews and Meta-Analyses (PRISMA) flow diagram is shown in Figure 1.

## Study Characteristics

All 6 included studies were hospital-based, with the exception of one multi-centric study, which had over 100 participants from 7 centers in 5 countries in SSA. The lowest age of patients was 7 years (1 month-18 years),^33^ which was the overall mean age of all children with all cancers in that study, and the highest age was 12 years (range 6-15 years).^17^ Majority of the children in the studies were male with an average of 76% (range 71%-86%). Age was reported in all of the studies.^3,17,31-34^ Characteristics of included studies are shown in Table 1.

**Table 1.** Characteristics of Included Studies.

Study ID	Number of participants	Country/setting	Study design/focus	Study period/median follow-up	Age	Sex (Number)
Chakumatha, 2020^31^	11	Malawi/Uni centric	Retrospective study	January 2017-December 2018	<18 years	NR
El-Mallawany et al, 2017^32^	21	Malawi/Uni centric	Retrospective study	December 2011-June 2013/13 months	Median 11.5 years (range 3.3-15.3)	M-71% F-29%
Schroeder, 2018^33^	06	Tanzania/Uni centric	Retrospective study	January 2010-August 2014	Overall mean age - 7 years (1 month-18 years)	NR
Togo, 2011^17^	07	Mali/Uni centric	Retrospective study	January 2005-December 2009/37 months	Mean 11.7 years (range 06-15)	M-86% F-14%
Traore, 2020^3^	106	Burkina Faso, Cameroon, Mali, Madagascar, Senegal/multicentric	Prospective study	October 2006-November 2012/30 months	Median-10 years, range (2-18)	M-75.5% F-24.5%
Yao, 2013^34^	07	Ivory Coast/Uni centric	Retrospective study	January 1995-December 2004	Median-8.4 years	M-71.4% F-28.6%

Two of the studies had a good quality overall rating^3,32^, one was fair^17^ and the remaining 3 had a poor overall rating.^31,33,34^ Results of studies with high Risk of Bias were not included in the analysis of the review, therefore, the results of 3 of the 6 studies were considered. Quality of the included studies is shown in Supplemental Table S2.

The clinical characteristics of the participants were described in 3 of the 6 studies,^3,17,32^ contrary to the other 3 studies,^31,33,34^ which did not report details of clinical characteristics. The most common clinical presentation was lymphadenopathy (>70%). And the sclerosing and lymphocyte-depleted were the most common and least common sub-types, respectively. Details of the characteristics of the participants in the included studies are given in Supplemental Table S3.

Disease staging was completed using the Ann Arbor stage classification^35^ as shown in Supplemental Table S4 and Figure S1.^35^ Stages were classified as stage I, stage II, stage III, and stage IV. Suffixes A and B were used depending on the absence or presence of B symptoms, respectively. Patients were further grouped depending on the prognosis at initial diagnosis: group 1 (favorable) and group 2 (unfavorable/advanced disease). Group 1 included stages I, II, IIB with no risk factors, and Group 2 included stages IIB, III, and IV with risk factors.^35^ However, most of the patients (about 81%) in the analysis belonged to group 2 as shown in Table 2.

**Table 2.** Summary of Characteristics of Studies Included in the Meta-Analysis.

study	Total number	Median age (years)	Median follow-up (months)	Quality of study (R.O.B)	regimen	diagnosis	Staging (%)/group (%)		Clinical characteristics (%)	Histological types
El-Mallawany, 2017^32^	21	11.5 (3.3-15.3)	13 (5-26)	Good (low)	ABVE/PC	Clinical-100% Pathology-86%	I/II- 52 III-29 1V-14 UNK-5	Group 1-52 Group 2-43	Abdominal mass-29 Mediastinal mass-14 lymphadenopathy -95 Cytopenia-10	NR
Togo, 2011^17^	07	11.7 (6-15)	37	Fair (moderate)	COPP/ABV	Clinical-100% Pathology-100%	IIB-71.4 III-28.6 Group 2-100		Erythrocyte sedimentation rate-85.7 Lymph nodes-100 LAD-71.4 Fever-42.8 Night sweats-28.6	NS-42.8 LR-28.6 MC-14.3 LD-14.3
Traore, 2020^3^	106	10 (2-18	30	Good (low)	COPP/ABV	Clinical-100% Pathology-100%	IA-2 IB-7 IIA-6 IIB-18 Group 1-14.2	IIIA-8 IIIB-47 IVA-3 IVB-15 Group 2-85.8	Erythrocyte sedimentation rate, lymphadenopathy, liver and spleen size measured in all patients.	NS-36 LR-16 MC-20 LD-2 UNK-26

Abbreviations: ROB, risk of bias; ABVE-PC, Adriamycin, Bleomycin, Vinblastine, Etoposide- Prednisone, Cyclophosphamide; COPP/ABV, Cyclophosphamide, Oncovin, Procarbazine, Prednisone- Adriamycin, Bleomycin, Vinblastine; NS, nodular sclerosing; LR, lymphocyte rich; MC, mixed cellularity; LD, lymphocyte depleted; UNK, unknown.

### Survival Outcomes

Supplemental Table S5 details the survival outcomes of all the included studies, whereas Table 3 shows details from the studies included in the meta-analysis. Only studies with a moderate or low risk of bias^3,17,32^ were included in the outcomes’ assessment.

**Table 3.** Summary of the Outcomes of the Studies Included in the Meta-Analysis.

Study ID	Number N	OS N (%)	CR N (%)	EFS N (%)	Deaths/treatment related deaths N (%)	TxA N (%)	Measurement time points (months)
El-Mallawany, 2017^32^	21	10 (48)	-	-	10 (48)/1 (5)	(5)	12
Togo, 2011^17^	07	5 (71.4)	5 (71.4)	-	2 (28.6)/2 (28.6)	(0)	37
Traore, 2020^3^	106	(82)	-	(67)	20 (18.9)/4 (3.77)	(0)	30

Abbreviations: OS, overall survival; CR, complete remission; EFS, event free survival; TxA, treatment abandonment.

Two of the studies with higher OS (82%^3^ and 71.4%^17^) had complete clinical examinations and staging done for all their patients with 100% histologically confirmed diagnoses compared to the other^32^ that had pathology done on only 86% of the patients diagnosed. The tests included physical examination, complete blood count, chest radiography, sedimentation rate, liver and renal functional tests and ultrasound scans. The 2 studies also utilized the COPP/ABV treatment regimen as compared to the other study that used ABVE/PC^32^ with a mortality rate of 48% as compared to 18.9%^3^ and 28.6%^17^ respectively. The treatment abandonment rate in one study was 5%^32^ as compared to the 2 studies, which were insignificant^3,17^ (Table 3). Details of the characteristics of the studies included in the meta-analysis are shown in Table 2.

**Figure 2.**
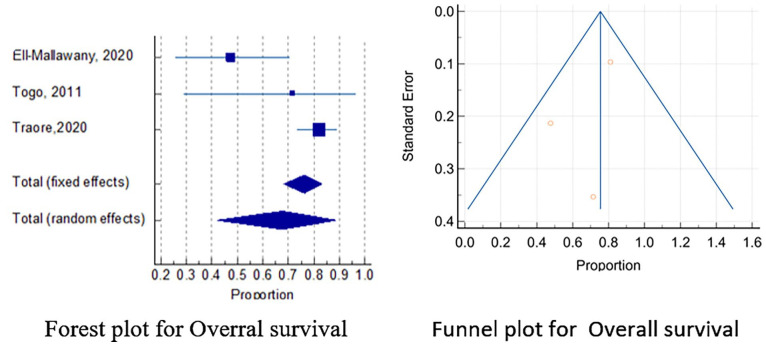
Meta-analysis plots for overall survival.

**Figure 3.**
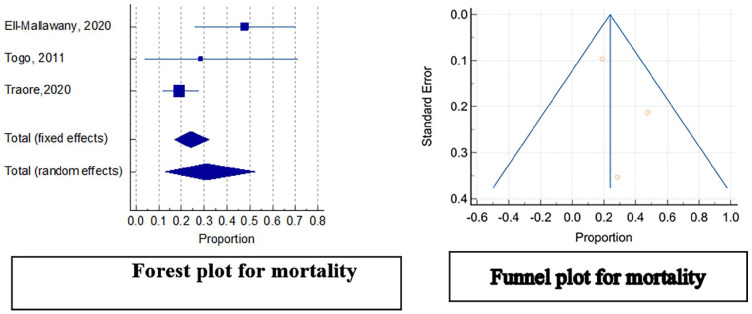
Meta-analysis plots for mortality.

**Figure 4.**
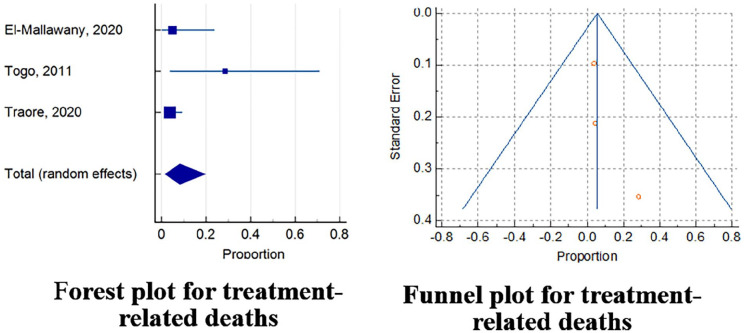
Meta-analysis plots for treatment-related deaths.

Results from the meta-analysis on percentages of the outcomes for chemotherapy-alone using the random effects method showed an overall estimate of 68% (95% CI: 42.103, 88.760) with *I*^2^ of 80.14%, *P* = 0.0065 for Overall Survival (Figure 2); 30.8% (95% CI: 12.862, 52.340) with *I*^2^ of 72.29%, *P* = 0.0271 for mortality (Figure 3); and 8.2% (95% CI: 1.423, 19.753) with *I*^2^ of 55.27%, *P* = 0.1069 treatment related deaths (Figure 4) in the 3 included studies.^3,17,32^ Further statistical output of the meta-analysis of overall survival, mortality and treatment related-mortality are illustrated in Supplemental Figures S2, S3, and S4 respectively.

Independently, for studies that had the same treatment regimen with histopathology and disease staging completed on all their patients,^3,17^ the OS that was observed was relatively high (71.4% and 82%).

### Secondary Outcomes

In 2 of the included studies, event-free survival at 30 months (Table 3) and a much lower Progression-free survival at 7 weeks (Supplemental Table S5) were reported: 67%^3^ and 9%,^31^ respectively. A relatively similar CR rate was reported in 2 of the studies as their primary outcome: 73% at 7 weeks^31^ (Supplemental Table S5), and 71.4% at 37 months^17^ (Table 3).

### Publication Bias

The funnel plots for all the assessed outcomes showed symmetrical plots with results evenly distributed, showing undetected publication bias (Figures 2-4). This was also illustrated by the results of Egger’s test as shown in the Supplemental Figures S2, S3, and S4 respectively.

### Grade Quality Assessment

The assessment was conducted on the following outcomes: OS and mortality, as they were considered critical outcomes as compared to EFS, PFS, and treatment abandonment, which were considered only important but not critical outcomes. Details are presented in Table 4. Overall, the certainty of the evidence was rated as very low for both outcomes assessed, as the results were imprecise but consistent and direct.

**Table 4.** GRADE Summary of Findings.

Study design/No. of studies	Risk of bias	inconsistency	indirectness	imprecision	Publication bias	Other considerations	Number of patients	importance	Certainty of Evidence
*Mortality (follow-up: range 12-37 months; Scale from: 18.9% to 48%)*									
Observational studies (3)	Not serious	Not serious	Not serious	Serious ^a^	Undetected	none	134	critical	⊕〇 〇〇 Very low
*Overall survival (follow-up: range 12-37 months; Scale from: 48% to 82%)*									
Observational studies (3)	Not serious	Not serious	Not serious	Serious ^a^	Undetected	none	134	critical	⊕〇 〇〇 Very low

a No confidence intervals given, small sample size.

## Discussion

Five studies were included in this review, and all were observational in nature. This was because the evidence around this subject was mostly from cancer registries in the various treatment centers, and this has been observed in other studies of pediatric cancers.^36,37^

### Summary of Findings

Studies included in the analysis had considerable statistical heterogeneity. However, it has been argued that the uncertainty of the use of this test with very few studies or small sample sizes is high and that although it is inevitable, the heterogeneity test (*I*^2^) may be irrelevant to the choice of analysis in this case.^38^ Furthermore, the *I*^2^ statistic test interpretation was designed in the context of analysis of randomized trials^29^ therefore it may not apply to observational studies. Some studies^33,34^ had incomplete data and some variations in their methodologies, which can affect the reliability of the conclusions. However, these were not included in the meta-analysis.

### Overall Survival and Mortality

The survival outcomes in this review are highly comparable to those reported elsewhere in developing countries.^11^ Mutyaba et al ^11^ conducted a study in Uganda among HL pediatric patients who received chemotherapy-only regimens and reported a 1-year overall survival rate of 67.5% and 30% mortality. However, OS observed in developed countries was significantly high: In the USA,^9^ an RCT conducted by the children’s cancer group compared treatment outcomes of patients on CT and chemotherapy and radiotherapy (CTR) in children with HL. They reported a 3-year overall survival of 99% and 98% respectively with a mortality of 4.9% for the entire study. This RCT conducted in the USA showed no survival benefits for radiation therapy.^39^ In another study in a middle-income country (Morocco) in Africa that intervened with CT, a 5-year OS of 88% and a mortality of 7.5% were reported. They utilized a multidisciplinary team approach, an abandonment prevention program, a uniform protocol-based therapy, a data collection system, and a twinning program with a developed country. These strategies improved their outcomes.

The survival outcomes in this review are most likely attributed to the following: late presentation of patients with advanced disease (the majority of the patients presented with advanced disease); variance in the treatment protocols used (the use of the COPP/ABV regimen seemed to be related to higher OS); inadequate provision of supportive care; the type of HL the patients had. Some studies had incomplete staging, meaning the disease staging was most likely underestimated, which would most likely affect the survival outcomes of HL in this review. However, treatment abandonment, which also affects overall survival, in this review was relatively insignificant. All patients had Classic HL, which has a fairly poor prognosis compared to NLPHL.^40^ Furthermore, the Nodular Sclerosing (NS) subtype, which was the most predominant type in this review (36.28%) has a worse prognosis compared to the mixed cellular (MC) type.^40^ This could also have contributed to mortality in this review. Based on the available evidence in this review, findings suggest that among children with HL, treatment with CT is relatively well tolerated and is a valuable treatment option in the absence of radiotherapy.

### Population Size

This review contained studies with small sample populations. This can be explained by probably the rarity of childhood HL,^4^ but also because the studies were conducted in the only cancer treatment centers in their respective countries. Patients from areas far from the treatment centers are challenged with access to treatment.^17^ This observation is consistent with findings from a recent review^41^ that noted that there were still few children cancer treatment centers in Africa. As elaborated by Togo et al,^17^ the long distance from treatment centers may partly explain the low numbers of patients presented at the health units or even the late diagnosis of patients made considering the low-income status of the population. Furthermore, in that review, data were obtained from several of population-based registries in SSA which had at least 70% coverage of their target population but had relatively low sample sizes: Kenya, Eldoret (2007-2011) had 282 patients, Ethiopia, Addis Ababa (2011-2013) had 160 patients, Mauritius (2003-2012) had 270 patients, the Gambia (2002-2011) had 194 patients, Niger, Niamey (2001-2009) had 175 patients. Similar findings were also observed from a national cancer registry in Uganda (2006-2009) a developing country in Africa which had 20 HL pediatric patients.^11^ Small sample sizes limit the generalizability of study findings and can limit the chances of a true association of factors.^42^

### Diagnosis and Staging

In the present review, it was noted that there was limited capacity in conducting the pathology of HL patients. To note, out of the 3 studies included in the analysis, only 2 studies had 100% of histology done on their patients^3,17^ whereas the other study^32^ had 86% of the patients pathologically confirmed. And there was no centralized review of histology results in one multicentric study.^3,17^ Studies that had a complete diagnosis (both clinically and histologically) and sufficient availability of drugs showed better survival outcomes.

There existed several differential diagnoses with similar characteristics like HL, which could lead to late diagnosis or misleading diagnosis if thorough diagnostics are not conducted. For instance, the common B-symptoms in HL are also typical in pulmonary TB, shortness of breath/airway obstruction which is usually caused by compression by the mediastinal mass could be easily confused with pneumonia, immunosuppression also in HIV, neutropenia, and anemia also very common in Malaria.^10,32^ Also, this problem is more likely explained by the resource limitedness in SSA to carry out adequate pathology and conduct other disease work-ups while also providing chemotherapy.^3^ This finding is similar to what is reported by other studies conducted in Africa, where treatment of HL is mainly provided by the government^10,39^ whose resources are at times limited. A review conducted on African studies on childhood HL, noted the challenges of correctly identifying HL in Africa as accessibility limitations to health services and ignoring the diagnosis of HL at the point of care due to a lack of efficient pathology service. These are also limitations that were noted in the studies included in this review.^3,17,32^ It was also noted that many hospital registers in Africa lacked vital information like staging.^10^ The problem of Late/misdiagnosis in this review can also explain the rate of patients with advanced disease at diagnosis. However, the rates of advanced disease observed in this review (66.4%) are Similar to findings in several African countries like South Africa (2001-2010) only 6.3% in stage 1, Uganda (2006-2009) 25% in stage 1 and Morocco (2004-2007) 54% in an advanced stage, contrary to what has been observed in the developed countries^43^ in the US centers 36% of patients presented with stages 3 and 4.

### Clinical Characteristics

Among clinical presentations of patients in this review, lymphadenopathy was the most common symptom (over 70%) which agreed with reports elsewhere.^40,44^ Roberts et al noted that mediastinal lymphadenopathy occurred in up to 66% of patients^40^ whereas Sherief et al in a study in Egypt observed lymphadenopathy as the most common presentation in the patients (96.6%).^44^

### Strengths of the Review

Notably, this review was carried out in line with the best practice guidelines of PRISMA and the Cochrane handbook for systematic reviews of interventions using validated methods to appraise the quality of studies included^19,45^ which ensured the internal validity of the results of the study. Also, pre-determined eligibility criteria were used to screen for eligible studies: a key characteristic of an SR.^46^ Furthermore, an optimal search of several databases, gray literature, pre-print servers, and reference lists was done to limit publication and selection bias, making sure that all eligible studies were included.^47^

The review considered all critical and important outcomes of the study and transparently graded the certainty of the evidence they presented, allowing an appropriate measure of the intervention to make appropriate recommendations for practice.^27,48^

### Limitations of the Review

Due to the nature of the subject under study, this review only included observational studies, of which available literature^49^ suggests that they provide a weak level of evidence for evaluating intervention effectiveness and are prone to a substantial source of bias. Studies in this review used a variety of treatment regimens in the management of HL. In this case, selection and information bias may originate from the difference in measurement of the intervention and use of different protocols in the studies.^50^ Although the included studies did not have a serious risk of bias as assessed by the NIH assessment tool in this review, they were limited by the fact that they had small sample sizes, which affect the precision of results by overestimating the treatment effect.^51^ However, the random effects model used in the meta-analysis gave more weight to these smaller studies. There was moderate to substantial heterogeneity between studies however this is usually the case when the number of studies involved in a review is small.^52^

### Limitations of the Evidence

The included studies were carried out in resource-limited countries where some limitations were encountered that may have affected the evidence produced in the studies. These included difficulty in access to the treatment centers,^17,32^ inability to carry out diagnostics like pathology, over-reliance on clinical diagnosis, shortage of trained staff like surgeons, and inadequate supportive care.^32^

## Conclusion

The GRADE certainty of the evidence for the assessed outcomes was rated as very low due to imprecision of results and because all evidence was from observational studies, therefore interpretation of results needs to be with caution because the actual effectiveness of chemotherapy alone in childhood HL in SSA may substantially be different from the estimate produced in this review. Notably, this review shows that optimal survival of children with HL in SSA can be achieved with the use of chemotherapy alone, however this is still very low as compared to developed countries. It also presents challenges that are faced during the management of childhood HL in SSA. It is important to recognize the role of early detection of HL and supportive care in the management of childhood HL.

### Implications for Practice

The OS in this review showed that chemotherapy alone may be well-established for the treatment of childhood HL, however, the rates were still low. Change in existing guidelines for the treatment of childhood HL may not be recommended based on the review’s results and the level of certainty of evidence. In the meantime, clinicians should continue utilizing and following the existing guidelines in the management of HL. Some studies noted challenges with the availability of resources,^3,17,31-33^ proper or complete documentation of cancer registries,^33^ staging and diagnosing HL,^32^ early detection and referrals of HL cases, and sufficient technical and support staff^17^ therefore, emphasis should be put on these to improve outcomes, reporting and measurement of OS in SSA. Furthermore, the author advocates for international collaborations between centers in developed and developing countries to improve infrastructure and professional development in SSA. This has shown improvement of OS in some other african countries like South Africa and Morocco.^39,53^

### Implications for Research

Three primary studies in this review^3,17,31^ reported high overall survival of patients on chemotherapy alone in SSA. To increase the certainty of results, further study in this area is recommended with results from well-designed and adequately powered large/multi-centric observational studies or RCTs if possible, utilizing the recommended staging and diagnosis methods for childhood HL. Notably, research should also focus on the identification of clinical factors that may affect the survival outcome of childhood HL on chemotherapy alone in SSA because the use of chemotherapy alone remains of interest because of the desire to limit late treatment sequelae of radiotherapy.

Further comprehensive studies on the utilization of chemotherapy-alone treatment should be undertaken to support the findings of this review and to demonstrate the survival rates of patients relative to those undergoing radiation therapy. It has been reported that OS differs depending on some factors. A study in a developed country observed several factors related to the disease, patient, and therapy like the presence of bulky disease and B-symptoms especially in the early disease stage, age of the patient and high ESR levels, chemotherapy regimen used, gender, and stage at diagnosis as some of the prognostic predictors of HL.^54^

## Supplemental Material

sj-docx-1-gph-10.1177_2333794X231223266 – Supplemental material for Childhood Hodgkin Lymphoma in Sub-Saharan Africa: A Systematic Review on the Effectiveness of the Use of Chemotherapy Alone Click here for additional data file. Supplemental material, sj-docx-1-gph-10.1177_2333794X231223266 for Childhood Hodgkin Lymphoma in Sub-Saharan Africa: A Systematic Review on the Effectiveness of the Use of Chemotherapy Alone by Hellen Mugarra Kabahweza and Angela Spencer in Global Pediatric Health

sj-docx-10-gph-10.1177_2333794X231223266 – Supplemental material for Childhood Hodgkin Lymphoma in Sub-Saharan Africa: A Systematic Review on the Effectiveness of the Use of Chemotherapy Alone Click here for additional data file. Supplemental material, sj-docx-10-gph-10.1177_2333794X231223266 for Childhood Hodgkin Lymphoma in Sub-Saharan Africa: A Systematic Review on the Effectiveness of the Use of Chemotherapy Alone by Hellen Mugarra Kabahweza and Angela Spencer in Global Pediatric Health

sj-docx-2-gph-10.1177_2333794X231223266 – Supplemental material for Childhood Hodgkin Lymphoma in Sub-Saharan Africa: A Systematic Review on the Effectiveness of the Use of Chemotherapy Alone Click here for additional data file. Supplemental material, sj-docx-2-gph-10.1177_2333794X231223266 for Childhood Hodgkin Lymphoma in Sub-Saharan Africa: A Systematic Review on the Effectiveness of the Use of Chemotherapy Alone by Hellen Mugarra Kabahweza and Angela Spencer in Global Pediatric Health

sj-docx-3-gph-10.1177_2333794X231223266 – Supplemental material for Childhood Hodgkin Lymphoma in Sub-Saharan Africa: A Systematic Review on the Effectiveness of the Use of Chemotherapy Alone Click here for additional data file. Supplemental material, sj-docx-3-gph-10.1177_2333794X231223266 for Childhood Hodgkin Lymphoma in Sub-Saharan Africa: A Systematic Review on the Effectiveness of the Use of Chemotherapy Alone by Hellen Mugarra Kabahweza and Angela Spencer in Global Pediatric Health

sj-docx-4-gph-10.1177_2333794X231223266 – Supplemental material for Childhood Hodgkin Lymphoma in Sub-Saharan Africa: A Systematic Review on the Effectiveness of the Use of Chemotherapy Alone Click here for additional data file. Supplemental material, sj-docx-4-gph-10.1177_2333794X231223266 for Childhood Hodgkin Lymphoma in Sub-Saharan Africa: A Systematic Review on the Effectiveness of the Use of Chemotherapy Alone by Hellen Mugarra Kabahweza and Angela Spencer in Global Pediatric Health

sj-docx-5-gph-10.1177_2333794X231223266 – Supplemental material for Childhood Hodgkin Lymphoma in Sub-Saharan Africa: A Systematic Review on the Effectiveness of the Use of Chemotherapy Alone Click here for additional data file. Supplemental material, sj-docx-5-gph-10.1177_2333794X231223266 for Childhood Hodgkin Lymphoma in Sub-Saharan Africa: A Systematic Review on the Effectiveness of the Use of Chemotherapy Alone by Hellen Mugarra Kabahweza and Angela Spencer in Global Pediatric Health

sj-docx-6-gph-10.1177_2333794X231223266 – Supplemental material for Childhood Hodgkin Lymphoma in Sub-Saharan Africa: A Systematic Review on the Effectiveness of the Use of Chemotherapy Alone Click here for additional data file. Supplemental material, sj-docx-6-gph-10.1177_2333794X231223266 for Childhood Hodgkin Lymphoma in Sub-Saharan Africa: A Systematic Review on the Effectiveness of the Use of Chemotherapy Alone by Hellen Mugarra Kabahweza and Angela Spencer in Global Pediatric Health

sj-docx-7-gph-10.1177_2333794X231223266 – Supplemental material for Childhood Hodgkin Lymphoma in Sub-Saharan Africa: A Systematic Review on the Effectiveness of the Use of Chemotherapy Alone Click here for additional data file. Supplemental material, sj-docx-7-gph-10.1177_2333794X231223266 for Childhood Hodgkin Lymphoma in Sub-Saharan Africa: A Systematic Review on the Effectiveness of the Use of Chemotherapy Alone by Hellen Mugarra Kabahweza and Angela Spencer in Global Pediatric Health

sj-docx-8-gph-10.1177_2333794X231223266 – Supplemental material for Childhood Hodgkin Lymphoma in Sub-Saharan Africa: A Systematic Review on the Effectiveness of the Use of Chemotherapy Alone Click here for additional data file. Supplemental material, sj-docx-8-gph-10.1177_2333794X231223266 for Childhood Hodgkin Lymphoma in Sub-Saharan Africa: A Systematic Review on the Effectiveness of the Use of Chemotherapy Alone by Hellen Mugarra Kabahweza and Angela Spencer in Global Pediatric Health

sj-docx-9-gph-10.1177_2333794X231223266 – Supplemental material for Childhood Hodgkin Lymphoma in Sub-Saharan Africa: A Systematic Review on the Effectiveness of the Use of Chemotherapy Alone Click here for additional data file. Supplemental material, sj-docx-9-gph-10.1177_2333794X231223266 for Childhood Hodgkin Lymphoma in Sub-Saharan Africa: A Systematic Review on the Effectiveness of the Use of Chemotherapy Alone by Hellen Mugarra Kabahweza and Angela Spencer in Global Pediatric Health
