# Supplementary material for: Childhood Hodgkin Lymphoma in Sub-Saharan Africa: A Systematic Review on the Effectiveness of the Use of Chemotherapy Alone
Source: Glob Pediatr Health. 2024 Jan 5;11:2333794X231223266. doi: 10.1177/2333794X231223266 (PMC10771044; doi:10.1177/2333794X231223266)
Supplement: sj-docx-10-gph-10.1177_2333794X231223266 – Supplemental material for Childhood Hodgkin Lymphoma in Sub-Saharan Africa: A Systematic Review on the Effectiveness of the Use of Chemotherapy Alone [file sj-docx-10-gph-10.1177_2333794X231223266.docx]

| **Study ID** | **participants** | **OS** | **CR** | **PFS** | **EFS** | **Death rate/**  **treatment related** | **Others**  **N (%)** | **Measurement**  **time points** |
| --- | --- | --- | --- | --- | --- | --- | --- | --- |
| Chakumatha, 2020^24^ | 11 | 9(82%) | 8(73%) | 1(9%) | - | 1(9%)/ 1(9%) | TxA 1(9%) | 7 weeks |
| El-Mallawany, 2017^25^ | 21 | 10(48%) | - | - | - | 10(48%) / 1(5%) | TxA 1(5%) | 12 months |
| Schroeder, 2018^26^ | 06 | 3(50%) | - | - | - | 2(33%)/NR | TxA 1(17%) | 2 years |
| Togo, 2011^16^ | 07 | 5(71.4%) | 5(71.4%) | - | - | 2(28.6%) / 2(28.6%) | TxA (0%) | 37 months |
| Traore, 2020^3^ | 106 | 82% | - | - | 67% | 20(18.9%) / 4(3.77%) | TxA (0%) | 36 months |
| Yao, 2013 ^27^ | 07 |  |  |  |  | 0% | Relapse-14.3%  LTF-85.7% | 12 months |

***Abbreviations: OS-overall survival; CR-complete remission; PFS-progression free survival; EFS-event free survival; TxA-treatment abandonment; LTF-loss to follow-up***

**Table S5: Summary of the outcomes of the included studies.**
