# Supplementary figures and images for: Childhood Hodgkin Lymphoma in Sub-Saharan Africa: A Systematic Review on the Effectiveness of the Use of Chemotherapy Alone
Source: Glob Pediatr Health. 2024 Jan 5;11:2333794X231223266. doi: 10.1177/2333794X231223266 (PMC10771044; doi:10.1177/2333794X231223266)

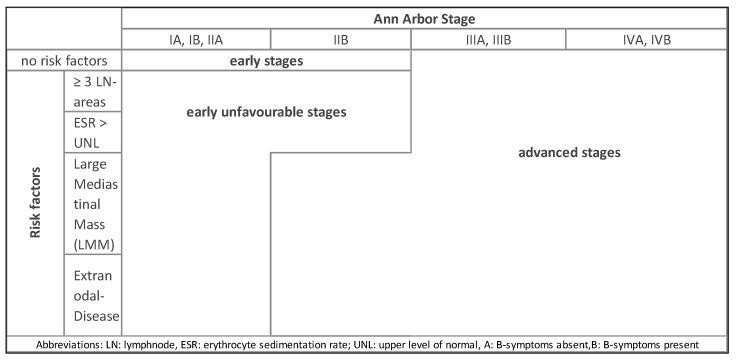


**Figure S1: Risk-stratified staging according to risk factors and Ann Arbor stage.**

Supplement: sj-docx-2-gph-10.1177_2333794X231223266 – Supplemental material for Childhood Hodgkin Lymphoma in Sub-Saharan Africa: A Systematic Review on the Effectiveness of the Use of Chemotherapy Alone [file sj-docx-2-gph-10.1177_2333794X231223266.docx]
