# Supplementary material for: Childhood Hodgkin Lymphoma in Sub-Saharan Africa: A Systematic Review on the Effectiveness of the Use of Chemotherapy Alone
Source: Glob Pediatr Health. 2024 Jan 5;11:2333794X231223266. doi: 10.1177/2333794X231223266 (PMC10771044; doi:10.1177/2333794X231223266)
Supplement: sj-docx-3-gph-10.1177_2333794X231223266 – Supplemental material for Childhood Hodgkin Lymphoma in Sub-Saharan Africa: A Systematic Review on the Effectiveness of the Use of Chemotherapy Alone [file sj-docx-3-gph-10.1177_2333794X231223266.docx]

| Variable for studies | | | study | | |
| --- | --- | --- | --- | --- | --- |
| Variable for total number of cases | | | total (n) | | |
| Variable for number of positive cases | | | OS | | |
| Study | Sample size | Proportion (%) | 95% CI | Weight (%) | |
|  |  |  |  | Fixed | Random |
| Ell-Mallawany, 2020 | 21 | 47.619 | 25.713 to 70.219 | 16.06 | 34.09 |
| Togo, 2011 | 7 | 71.429 | 29.042 to 96.331 | 5.84 | 24.68 |
| Traore,2020 | 106 | 82.075 | 73.432 to 88.849 | 78.10 | 41.22 |
| Total (fixed effects) | 134 | 76.274 | 68.258 to 83.119 | 100.00 | 100.00 |
| Total (random effects) | 134 | 67.811 | 42.103 to  88.760 | 100.00 | 100.00 |
| \| **Test for heterogeneity** \| \| **Publication Bias** \| \| \| --- \| --- \| --- \| --- \| \| Egger's test \| \| \| Q \| 10.0697 \| Intercept \| -2.7938 \| \| DF \| 2 \| 95% CI \| -36.8964 to 31.3088 \| \| Significance level \| P = 0.0065 \| Significance level \| P = 0.4872 \| \| I^2^ (inconsistency) \| 80.14% \| Begg's test \| \| \| 95% CI for I^2^ \| 37.29 to 93.71 \| Kendall's Tau \| -0.3333 \| \|  \|  \| Significance level \| P = 0.6015 \| | | | | | |

Figure S2: Output from MedCalc statistical software for the meta-analysis on Overall Survival.
