# Supplementary material for: Childhood Hodgkin Lymphoma in Sub-Saharan Africa: A Systematic Review on the Effectiveness of the Use of Chemotherapy Alone
Source: Glob Pediatr Health. 2024 Jan 5;11:2333794X231223266. doi: 10.1177/2333794X231223266 (PMC10771044; doi:10.1177/2333794X231223266)
Supplement: sj-docx-4-gph-10.1177_2333794X231223266 – Supplemental material for Childhood Hodgkin Lymphoma in Sub-Saharan Africa: A Systematic Review on the Effectiveness of the Use of Chemotherapy Alone [file sj-docx-4-gph-10.1177_2333794X231223266.docx]

| Variable for studies | | | studies | | |
| --- | --- | --- | --- | --- | --- |
| Variable for total number of cases | | | total | | |
| Variable for number of positive cases | | | cases | | |
| Study | Sample size | Proportion (%) | 95% CI | Weight (%) | |
|  |  |  |  | Fixed | Random |
| El-Mallawany 2017 | 21 | 47.619 | 25.713 to 70.219 | 16.06 | 33.68 |
| Togo 2011 | 7 | 28.571 | 3.669 to 70.958 | 5.84 | 22.06 |
| Traore 20208 | 106 | 18.868 | 11.923 to 27.625 | 78.10 | 44.26 |
| Total (fixed effects) | 134 | 23.901 | 17.034 to 31.931 | 100.00 | 100.00 |
| Total (random effects) | 134 | 30.751 | 12.862 to 52.340 | 100.00 | 100.00 |
| \| **Test for heterogeneity** \| \| **Publication bias** \| \| \| --- \| --- \| --- \| --- \| \| Q \| 7.2176 \| Egger's test \| \| \| DF \| 2 \| Intercept \| 2.4177 \| \| Significance level \| P = 0.0271 \| 95% CI \| -25.7452 to 30.5805 \| \| I^2^ (inconsistency) \| 72.29% \| Significance level \| P = 0.4724 \| \| 95% CI for I^2^ \| 6.45 to 91.79 \| Begg's test \| \| \|  \|  \| Kendall's Tau \| 0.3333 \| \|  \|  \| Significance level \| P = 0.6015 \| | | | | | |

Figure S3: Output from MedCalc statistical software for the meta-analysis on mortality
