# Supplementary material for: Childhood Hodgkin Lymphoma in Sub-Saharan Africa: A Systematic Review on the Effectiveness of the Use of Chemotherapy Alone
Source: Glob Pediatr Health. 2024 Jan 5;11:2333794X231223266. doi: 10.1177/2333794X231223266 (PMC10771044; doi:10.1177/2333794X231223266)
Supplement: sj-docx-5-gph-10.1177_2333794X231223266 – Supplemental material for Childhood Hodgkin Lymphoma in Sub-Saharan Africa: A Systematic Review on the Effectiveness of the Use of Chemotherapy Alone [file sj-docx-5-gph-10.1177_2333794X231223266.docx]

| Variable for studies | | | study ID | | |
| --- | --- | --- | --- | --- | --- |
| Variable for total number of cases | | | total | | |
| Variable for number of positive cases | | | number_of_deaths | | |
| Study | Sample size | Proportion (%) | 95% CI | Weight (%) | |
|  |  |  |  | Fixed | Random |
| Ell-Mallwany, 2020 | 21 | 4.762 | 0.120 to 23.816 | 16.06 | 31.71 |
| Togo, 2011 | 7 | 28.571 | 3.669 to 70.958 | 5.84 | 17.29 |
| Traore, 2020 | 106 | 3.774 | 1.038 to 9.381 | 78.10 | 51.00 |
| Total (fixed effects) | 134 | 5.533 | 2.352 to 10.790 | 100.00 | 100.00 |
| Total (random effects) | 134 | 8.180 | 1.423 to 19.753 | 100.00 | 100.00 |
| \| **Test for heterogeneity** \| \| **Publication bias** \| \| \| --- \| --- \| --- \| --- \| \| Egger's test \| \| \| Q \| 4.4715 \| Intercept \| 2.3446 \| \| DF \| 2 \| 95% CI \| -11.3852 to 16.0744 \| \| Significance level \| P = 0.1069 \| Significance level \| P = 0.2749 \| \| I^2^ (inconsistency) \| 55.27% \| Begg's test \| \| \| 95% CI for I^2^ \| 0.00 to 87.23 \| Kendall's Tau \| 1.0000 \| \|  \|  \| Significance level \| P = 0.1172 \| | | | |  |  |

Figure S4: Output from MedCalc statistical software for the meta-analysis on treatment-related deaths.
