# Supplementary material for: Childhood Hodgkin Lymphoma in Sub-Saharan Africa: A Systematic Review on the Effectiveness of the Use of Chemotherapy Alone
Source: Glob Pediatr Health. 2024 Jan 5;11:2333794X231223266. doi: 10.1177/2333794X231223266 (PMC10771044; doi:10.1177/2333794X231223266)
Supplement: sj-docx-6-gph-10.1177_2333794X231223266 – Supplemental material for Childhood Hodgkin Lymphoma in Sub-Saharan Africa: A Systematic Review on the Effectiveness of the Use of Chemotherapy Alone [file sj-docx-6-gph-10.1177_2333794X231223266.docx]

| **# 1** | "Hodgkin Disease"[Mesh] OR “Hodgkin Lymphoma”[Title/Abstract] OR “Hodgkin's Disease”[Title/Abstract] OR “Hodgkin's Lymphoma”[Title/Abstract] OR “Hodgkins Lymphoma”[Title/Abstract] OR “Hodgkins Disease”[Title/Abstract] | **70,995** |
| --- | --- | --- |
| **#2** | **"Drug Therapy"[Mesh] OR “Drug Therap*”**[Title/Abstract] **OR Chemotherap***[Title/Abstract] | **1,772,720** |
| **#3** | "Africa South of the Sahara"[Mesh] OR “Sub-Saharan Africa” OR “Subsaharan Africa” OR Cameroon OR “Central African Republic” OR chad OR Congo OR “Democratic Republic of the Congo” OR “Equatorial Guinea” OR Gabon OR “Sao Tome and Principe” OR Burundi OR Djibouti OR Eritrea OR Ethiopia OR Kenya OR Rwanda OR Somalia OR “South Sudan” OR Sudan OR Tanzania OR Uganda OR Angola OR Botswana OR Eswatini OR Lesotho OR Malawi OR Mozambique OR Namibia OR “South Africa” OR Zambia OR Zimbabwe OR Benin OR “Burkina Faso” OR “Cabo Verde” OR “Cote d'Ivoire” OR Gambia OR Ghana OR Guinea OR “Guinea-Bissau” OR Liberia OR Mali OR Mauritania OR Niger OR Nigeria OR Senegal OR “Sierra Leone” OR Togo | **608,674** |
| **#4** | infan*[tw] OR child*[tw] OR adolescen*[tw] OR pediatric*[tw] OR paediatric*[tw] OR pube*[tw] OR juvenil*[tw] OR school*[tw] OR newborn*[tiab] OR new-born*[tiab] OR neo-nat*[tiab] OR neonat*[tiab] OR premature*[tiab] OR pre-mature*[tiab] OR baby[tiab] OR babies[tiab] OR toddler*[tiab] OR youngster*[tiab] OR preschool*[tiab] OR kindergart*[tiab] OR kid[tiab] OR kids[tiab] OR playgroup*[tiab] OR play-group*[tiab] OR playschool*[tiab] OR prepube*[tiab] OR preadolescen*[tiab] OR junior high*[tiab] OR highschool*[tiab] OR senior high[tiab] OR young people*[tiab] OR minors[tiab] | **4,808,044** |
| **#5** | **#1 AND #2 AND #3 AND #4** | **58** |

Table S1: Search strategy of MEDLINE via PUBMED (14/DEC/2021). Applied filters for publication year (2000/January – 2022/December) and a Search block for children.
