# Supplementary material for: Childhood Hodgkin Lymphoma in Sub-Saharan Africa: A Systematic Review on the Effectiveness of the Use of Chemotherapy Alone
Source: Glob Pediatr Health. 2024 Jan 5;11:2333794X231223266. doi: 10.1177/2333794X231223266 (PMC10771044; doi:10.1177/2333794X231223266)
Supplement: sj-docx-7-gph-10.1177_2333794X231223266 – Supplemental material for Childhood Hodgkin Lymphoma in Sub-Saharan Africa: A Systematic Review on the Effectiveness of the Use of Chemotherapy Alone [file sj-docx-7-gph-10.1177_2333794X231223266.docx]

| Study | Q1 | Q2 | Q3 | Q4 | Q5 | Q6 | Q7 | Q8 | Q9 | Q10 | Q11 | Q12 | Q13 | Q14 | Overall |
| --- | --- | --- | --- | --- | --- | --- | --- | --- | --- | --- | --- | --- | --- | --- | --- |
| Chakumatha, 2020 | Y | Y | Y | Y | NR | Y | Y | N | N | N | Y | NA | Y | N | Poor |
| El-Mallawany, 2017 | Y | Y | Y | Y | NR | Y | Y | Y | Y | Y | Y | NA | Y | Y | good |
| Schroeder, 2018 | Y | N | Y | Y | NR | Y | Y | N | N | N | Y | NA | Y | Y | Poor |
| Togo, 2011 | Y | Y | Y | Y | NR | Y | Y | Y | Y | Y | Y | NA | Y | N | Fair |
| Traore, 2020 | Y | Y | Y | Y | NR | Y | Y | Y | Y | Y | Y | NA | Y | Y | good |
| Yao, 2013 | Y | Y | Y | Y | NR | Y | Y | N | NR | Y | Y | NA | N | N | Poor |

***Abbreviations****:* ***Q****: question,* ***Y****: yes;* ***N****: no;* ***NA****: not applicable;* ***NR****: not reported*

Table S2: Methodological quality assessment.
