# Supplementary material for: Childhood Hodgkin Lymphoma in Sub-Saharan Africa: A Systematic Review on the Effectiveness of the Use of Chemotherapy Alone
Source: Glob Pediatr Health. 2024 Jan 5;11:2333794X231223266. doi: 10.1177/2333794X231223266 (PMC10771044; doi:10.1177/2333794X231223266)
Supplement: sj-docx-8-gph-10.1177_2333794X231223266 – Supplemental material for Childhood Hodgkin Lymphoma in Sub-Saharan Africa: A Systematic Review on the Effectiveness of the Use of Chemotherapy Alone [file sj-docx-8-gph-10.1177_2333794X231223266.docx]

| **Study ID** | **Diagnosis criteria** | | **clinical characteristics** | **staging** | **Histology sub-types** | **Drug regimen used** |
| --- | --- | --- | --- | --- | --- | --- |
|  | **histology** | **clinical diagnosis** |  |  |  |  |
| Chakumatha, 2020 ^24^ | 8 (73%) | 3 (27%) | NR | NR | NR | NR |
| El-Mallawany, 2017 ^25^ | 18 (86%) | 3 (14%) | Abdominal mass – 6(29%)  Peripheral LAD- 20(95%)  Mediastinal mass-3(14%)  Cytopenias-2(10%) | Stage I/II –11 (52%)  Stage III – 6 (29%)  Stage IV – 3 (14%) | NR | ABVE-PC |
| Schroeder, 2018 ^26^ | HL specific –NR. | HL specific –NR | NR | NR | NR | NR |
| Togo, 2011 ^16^ | 7  (100%) | 7  (100%) | Cervical LAD- 71.4%  Lymph nodes-100%  Fever- 3(42.8%)  Night sweats-2(28.6%)  Sedimentation rate- 6(85.7%) | IIB- 5(71.4%), IIIB – 2(28.6%)  All patients were in group 2 | Scleronodular -3(42.8%)  Lymphocyte-rich-2(28.6%)  Mixed cellularity-1(14.3%)  Lymphocyte-depleted-1(14.3%) | COPP-ABV |
| Traore, 2020 ^3^ | 106  (100%) | 106 (100%) | LAD,  Liver and spleen size,  Erythrocyte sedimentation rate, chest-abdominal computed topography on all patients | Stage IA – 2  Stage IB – 7  Stage IIA – 6  Stage IIB – 18  Stage IIIA – 8  Stage IIIB – 47(44%)  Stage IVA – 3  Stage IVB- 15  Group 1-15  Group 2- 91 | Nodular sclerosis – 38(36%)  Mixed cellularity – 21(20%)  Lymphocyte-rich – 17(16%)  Lymphocyte-depleted – 2(2%)  Unknown type – 28(26%) | COPP-ABV |
| Yao, 2013 ^27^ | NR | NR | NR | ND | ND | NR |

Abbreviations: NR: not reported, ND: not done, LAD: lymphadenopathy, HL: Hodgkin Lymphoma, B: B symptoms present, ABVE-PC: Adriamycin, Bleomycin, Vinblastine, Etoposide- Prednisone, Cyclophosphamide, COPP-ABV: Cyclophosphamide, Oncovin, Procarbazine, Prednisone- Adriamycin, Bleomycin, Vinblastine

Table S3: Characteristics of participants in the included studies.
