# Supplementary material for: Childhood Hodgkin Lymphoma in Sub-Saharan Africa: A Systematic Review on the Effectiveness of the Use of Chemotherapy Alone
Source: Glob Pediatr Health. 2024 Jan 5;11:2333794X231223266. doi: 10.1177/2333794X231223266 (PMC10771044; doi:10.1177/2333794X231223266)
Supplement: sj-docx-9-gph-10.1177_2333794X231223266 – Supplemental material for Childhood Hodgkin Lymphoma in Sub-Saharan Africa: A Systematic Review on the Effectiveness of the Use of Chemotherapy Alone [file sj-docx-9-gph-10.1177_2333794X231223266.docx]

| **Stage** | **Explanation** |
| --- | --- |
| I | Involvement of one lymph node region or a single localized involvement outside the lymphatic system |
| II | Involvement of two or more lymph node regions on the same side of the diaphragm or localized involvement outside the lymphatic system and lymph node regions on the same side of the diaphragm |
| III | Involvement of two or more lymph node regions or organs outside the lymphatic system on both sides of the diaphragm |
| IV | Diffuse or disseminated infestation of one or more extra lymphatic organs with or without infestation of lymphoid tissue |
| A | No B-symptoms |
| B | B symptoms: fever, drenching night sweats, and/or unexplained loss of body weight >10% within the preceding 6 months. |

**Table S4: Ann Arbor classification for Hodgkin’s lymphoma.**
